# Measuring the Impact of AI on Report-Drafting Efficiency in Chest Computed Tomography Interpretation: Retrospective Analysis

**DOI:** 10.2196/77967

**Published:** 2026-03-27

**Authors:** Weiqi Liu, You Wu, Wei Yu, Mark J Bittle, Zhuozhao Zheng, Hadi Kharrazi

**Affiliations:** 1 Department of Health Policy and Management Bloomberg School of Public Health Johns Hopkins University Baltimore, MD United States; 2 Department of Research Sophmind Technology (Beijing) Co. Ltd Beijing China; 3 Institute for Hospital Management School of Medicine Tsinghua University Beijing China; 4 Department of Radiology Beijing Anzhen Hospital Capital Medical University Beijing China; 5 Department of Radiology Beijing Tsinghua Changgung Hospital, School of Clinical Medicine Tsinghua University Beijing China

**Keywords:** lung nodule, chest computed tomography, radiology, artificial intelligence, AI, efficiency, clinical workflow evaluation

## Abstract

**Background:**

Artificial intelligence (AI), particularly deep learning, has shown promise in enhancing medical image interpretation and improving radiologists’ efficiency. In China, growing imaging demand and workforce shortages have placed increasing pressure on radiology services. However, evidence on the operational impact of AI on reporting efficiency remains limited.

**Objective:**

This study aimed to evaluate the effect of an AI system on radiologists’ reporting efficiency by examining changes in report-drafting time for lung nodule diagnosis in chest computed tomography (CT) images.

**Methods:**

We analyzed 185,044 chest CT reports from Beijing Anzhen and Tsinghua Changgung Hospitals (2018-2023) using a difference-in-differences design with nonequivalent comparison groups. Report-drafting time before, immediately after, and up to 2 years following AI implementation was compared, adjusting for radiologist gender, seniority, and years of working experience.

**Results:**

The pooled analysis showed a modest overall increase of 0.86 minutes (95% CI 0.14 to 1.57). However, this masked substantial heterogeneity between hospitals due to differing implementation timelines. In the first year after AI deployment, Tsinghua Changgung Hospital experienced a nonsignificant increase of 0.90 minutes (95% CI –0.28 to 2.08). In contrast, at Beijing Anzhen Hospital, the AI-assisted group exhibited an absolute reduction of 0.76 minutes by the first year and a further 1.83-minute reduction by the second year (an approximate 28% time saved vs baseline), while the control group remained stable over time. Using a difference-in-differences framework, this corresponded to a 2.66-minute relative improvement compared with the counterfactual trend (*P*<.001).

**Conclusions:**

AI-assisted lung nodule diagnosis may initially increase report-drafting time due to adaptation and workflow adjustment. Sustained, meaningful efficiency gains were heterogeneous and observed at only 1 of the 2 study sites, indicating that long-term impacts are strongly contingent on site-specific implementation dynamics, learning curves, and local context.

## Introduction

Artificial intelligence (AI) can imitate human intelligence, perform tasks, and make progressive enhancements based on amassed data [1]. Particularly, the application of deep learning technology in medical image segmentation and recognition has brought revolutionary changes to the operation of imaging services [2]. The increasing adoption of AI has the potential to alter the working conditions of radiologists [3].

The rapid evolution of radiological modalities has resulted in a substantial increase in the volume of medical images. While this progress has numerous advantages, it has significantly increased radiologists’ workloads [4,5]. For example, workplace burnout could weaken radiologists’ perceived competence [6], and occupational fatigue may compromise radiologists’ diagnostic accuracy, thereby adversely affecting patient care and treatment outcomes [7]. Therefore, it is critical to explore how AI systems can enhance the efficiency of radiologists for more effective applications in clinical practice.

Globally, the scarcity of physicians persists due to the substantial professional requirements and protracted talent development time. This challenge is exacerbated in China, where high patient demand contrasts with inadequate investments in medical education, relatively low remuneration for health care practitioners, and enduring disproportions in medical resource allocation [8]. AI stands as a pivotal technological approach that could potentially alleviate the scarcity of medical resources in China. Furthermore, supportive governmental policies are poised to contribute significantly to the advancement of AI within the Chinese health care sector [9].

The impact of AI systems on the operational efficiency of radiologists remains a topic of uncertainty [10]. Within the medical and scientific communities, there is a prevailing belief that the rapid development of AI in health care will lead to a transformative paradigm shift in medical imaging. Enhancing operational efficiency has been deemed part of the AI’s paradigm shift in medical imaging [11]; however, empirical evidence is limited and sometimes conflicting [12,13]. For example, Yi et al [14] unveiled that the implementation of an AI system for breast cancer detection resulted in a substantial reduction in reporting time. Stephan et al [15] reported improved efficiency in generating dental radiology reports, while Zia et al [16] found that implementing an AI system for intracranial hemorrhage detection led to longer turnaround time. Nonetheless, it is essential to acknowledge that these findings may not provide a definitively representative picture, primarily due to the limited sample sizes of such studies [12]. Additionally, the results of these studies often do not provide a comprehensive understanding of the enduring implications of AI system adoption in medical imaging, as the study periods have been relatively brief [12].

This real-world study was conducted in 2 large hospitals in China, using data spanning multiple years and the difference-in-differences (DID) methodology. The study assesses the alterations in report-drafting time for lung nodule diagnosis in computed tomography (CT) scans after AI adoption. Results of this study can inform the radiological community on the prospective advantages and challenges associated with AI integration in medical imaging.

## Methods

### Study Population

**Figure 1:**
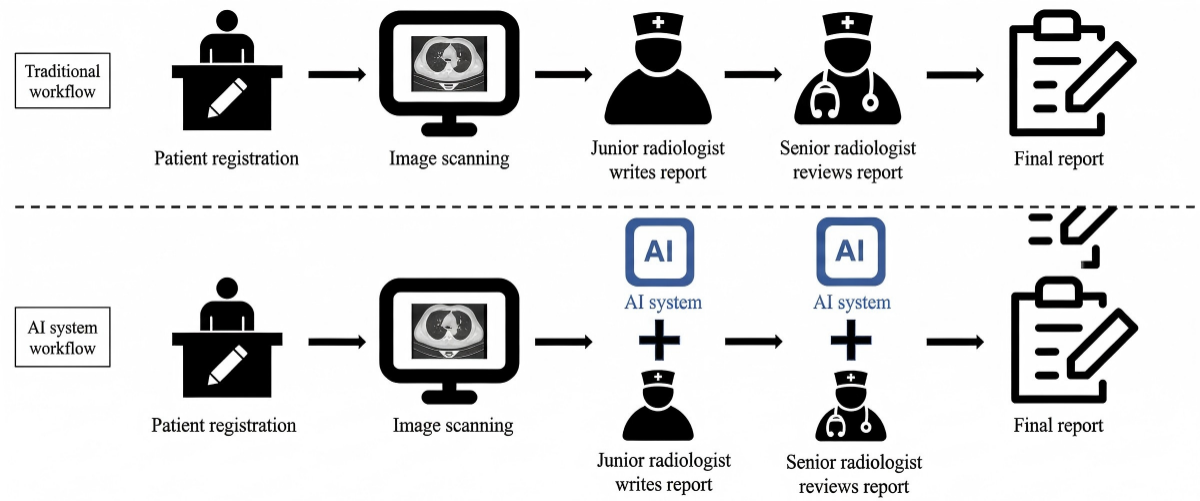
Traditional vs artificial intelligence (AI)–assisted workflows in the radiology departments of the 2 hospitals in this study.

This study was conducted at 2 tertiary health care facilities situated in Beijing, China: Beijing Anzhen Hospital and Tsinghua Changgung Hospital. Figure 1 illustrates the diagnostic process in the radiology departments both before and after the introduction of the AI system. In the traditional workflow, before the AI system’s implementation, the typical process involved a junior radiologist writing a diagnostic report after each patient underwent a CT examination. Subsequently, a senior radiologist would review and finalize this report before it was released to clinicians and patients. With the integration of the AI system, both junior and senior radiologists can use the findings of the AI systems as diagnostic aids. The AI system will automatically identify and analyze lung nodules in imported chest CT images. Junior and senior radiologists can reference the diagnostic results provided by the AI system during their diagnostic process and ultimately complete their diagnostic reports. Case assignment follows a randomized queue-based workflow in both hospitals, without systematic allocation of more complex cases to more experienced radiologists.

The lung nodule AI systems implemented at Beijing Anzhen Hospital and Tsinghua Changgung Hospital were developed and integrated by Care.ai [17] (Yitu, integrated into Deepwise’s framework in 2021) and Dr.Wise (Deepwise) [18]. Both systems followed a standardized reporting workflow and user interface, and no vendor change occurred during the study period. Both AI systems were approved by the National Medical Products Administration and provided comparable functionalities for lung nodule detection and reporting assistance. These AI systems were introduced at Beijing Anzhen Hospital in January 2019 and at Tsinghua Changgung Hospital in June 2021. The deployment phase, which involved installation and system integration, spanned approximately 1 month. Subsequently, an additional 3-month period was required to attain optimal and seamless system functionality. Multimedia Appendix 1 illustrates the features and capabilities of the AI-assisted diagnostic system as shared with radiologists. This system demonstrates the capability to automatically detect lung nodules; assess their quantity, dimensions, and precise locations; and subsequently generate corresponding diagnostic reports. Multimedia Appendices 2 and 3 illustrate the detailed operational workflow of the AI system and show that the AI system use processes in the 2 hospitals are essentially identical.

We analyzed the CT report-drafting time for individual patients from April 2018 to March 2022 at these 2 hospitals. Data for the following periods were excluded from the analysis: January 2019 to March 2019, January 2020 to August 2020, and April 2021 to August 2021, due to the potential impact of the COVID-19 pandemic, AI system integration, and the Spring Festival holiday on patterns of health seeking behavior in China [19]. Due to the considerable volatility in the night shift patterns of radiologists, this study exclusively incorporated day shift reports for analysis. To mitigate potential confounding factors related to workforce turnover, our analysis exclusively examined radiology reports written by the same group of radiologists who had generated reports both before and after the introduction of the AI system.

### Data Collection

Using the University of California, San Francisco calculator [20], sample size calculations indicated a necessary sample of 12 to detect an effect size of 1 (difference in minutes between AI-assisted and conventional report-drafting time) with a SD of 0.5, ensuring 80% power at an α level of .05 (2-tailed). With nearly 100,000 reports generated at each hospital during the study period, the sample markedly surpassed requirements.

The data collection procedure encompassed the extraction of all junior radiologist report-drafting time records from the Radiology Information System and Picture Archiving and Communication System of both hospitals throughout the specified sampling period. The collected dataset included various patient-related details, such as age, sex, source of referral, examination specifics, examination time, reporting time by both junior and senior radiologists, and the identities of the reporting radiologists.

The report-drafting time analyzed in this study reflects the first-round interpretation performed by junior radiologists. The primary outcome of interest, report-drafting time, was defined as the duration between loading the CT images and clicking the Submit button on the report form [21]. To ensure data quality, we conducted a detailed assessment of extreme values. Report-drafting time of less than 0.5 minutes or more than 40 minutes—together representing approximately 5% of all observations (short drafting time: 6287/195,020, 3.2%; long drafting time: 3689/195,020, 1.9%)—were excluded, as such values most likely reflected system artifacts or nonclinical interruptions (idle time) rather than true behaviors. To ensure data integrity, a 2-investigator workflow was used. One extracted data from the reporting systems, applied predefined inclusion and exclusion criteria, and calculated report-drafting times. A second investigator independently verified the processed dataset to ensure accuracy, completeness, and consistency.

### Statistical Analysis

This study used a DID design, which involved a real-world retrospective assessment of the outcome within an experimental and a comparison group. The experimental group in our study comprised chest CT images interpreted using the AI-aided diagnostic system following its implementation, while the comparison group included other CT images that were not affected by the introduction of the AI system.

Although the intervention group focused on chest CT images and the control group included other CT images (eg, abdominal, pelvic, and head CT), all examinations followed the same radiology workflow within each institution. This institutional uniformity ensured that reporting procedures were comparable, minimizing systematic bias unrelated to the AI intervention.

Before using the DID methodology, an assessment was conducted to evaluate the parallel trend assumption. This assumption necessitates that the trends exhibited by both the experimental and comparison groups remain consistent before the intervention is introduced [22]. The DID analysis used an ordinary least squares regression model, as shown in equation 1.




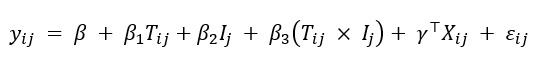




where the dependent variable (*y_ij_*) denotes the report-drafting time for case *i* interpreted by radiologist j. *Tij* indicates AI use (1 if the AI-assisted system was used, 0 otherwise), and *I_j_* denotes group assignment (1 for the experimental group and 0 for the comparison group). The interaction term *T_ij_*×*I_j_* captures the DID estimator of interest. *X_ij_* represents a vector of covariates, including radiologist sex, professional title rank, and years of working experience. SEs were clustered at the radiologist level to account for within-radiologist correlation. Models included hospital fixed effects to control for time-invariant institutional differences, as well as calendar quarter fixed effects to account for common seasonal and temporal shocks. We further tested for effect modification by radiologist seniority by adding AI-seniority interaction terms to the main model.

In this analysis, a significance level of *P*<.05 was used to determine statistical significance. The statistical analyses were conducted using Stata (version 15.1; StataCorp LLC) and R (version 4.4.1; R Foundation for Statistical Computing).

### Ethical Considerations

This study received ethics approval from the institutional review board (IRB) of Tsinghua Changgung Hospital (23352-4-01) and from the IRB of Beijing Anzhen Hospital (2023078X). Additional IRB approval was also granted by the Johns Hopkins Bloomberg School of Public Health (FWA 00000287). The IRB waived the requirement for informed consent, as this study involved secondary analysis of retrospective, noninterventional data originally collected for operational purposes. According to institutional policy, individual consent is not required for such analyses if the data are fully deidentified and used exclusively for quality improvement or research purposes. No compensation was provided to the participants in this retrospective study.

All data analyzed in this study were fully deidentified before access and analysis, with no personally identifiable information or protected health information included (sample data for review in Multimedia Appendix 4). Data were stored on secure institutional servers with access restricted to authorized study personnel only. This study does not contain any identifiable images or multimedia materials of individual participants. All figures and tables are based on aggregated, anonymized data.

## Results

The study incorporated 185,044 CT images that met the predefined criteria. Table 1 provides an overview of the demographic characteristics of patients from both the experimental and comparison groups, before and after the introduction of the AI system. Beijing Anzhen Hospital accounted for a larger proportion of patients after the AI launch due to its earlier implementation. The sex ratio between the experimental and comparison groups was comparable, but there were slightly more male patients than female patients in both groups. The source of patients varied slightly based on medical needs, with outpatient clinics contributing the largest number of patients.

**Table 1:** Patient characteristics before and after the artificial intelligence (AI) launch in experimental and control groups.

			Overall (n=185,044), n (%)		Before AI launch (n=93,870)					After AI launch (n=91,174)		
					Control group (n=55,592), n (%)		Experimental group (n=38,278), n (%)		Control group (n=50,751), n (%)			Experimental group (n=40,423), n (%)
**Age (years)**												
	<18	1665 (0.9)		676 (1.2)		253 (0.7)		424 (0.8)			312 (0.8)	
	18-54	72,493 (39.4)		19,513 (35.4)		16,711 (44.1)		18,703 (37.0)			17,566 (43.6)	
	>55	109,712 (59.7)		34,939 (63.4)		20,910 (55.2)		31,418 (62.2)			22,445 (55.7)	
**Sex**												
	Female	85,216 (46.1)		25,734 (46.3)		17,964 (46.9)		23,846 (47.0)			17,672 (43.7)	
	Male	99,828 (53.9)		29,858 (53.7)		20,314 (53.1)		26,905 (53.0)			22,751 (56.3)	
**Hospital**												
	Beijing Anzhen Hospital	97,366 (52.6)		23,163 (41.7)		7880 (20.6)		36,009 (71.0)			30,314 (75.0)	
	Tsinghua Changgung Hospital	87,678 (47.4)		32,429 (58.3)		30,398 (79.4)		14,742 (29.0)			10,109 (25.0)	
**Patient source**												
	Emergency room	57,687 (31.2)		14,946 (26.9)		8731 (22.8)		18,284 (36.0)			15,726 (38.9)	
	Inpatient	38,675 (20.9)		17,215 (31.0)		5684 (14.8)		11,084 (21.8)			4692 (11.6)	
	Outpatient	81,655 (44.1)		23,364 (42.0)		19,686 (51.4)		21,267 (41.9)			17,338 (42.9)	
	Physical exam	7027 (3.8)		67 (0.1)		4177 (10.9)		116 (0.2)			2667 (6.6)	

**Figure 2:**
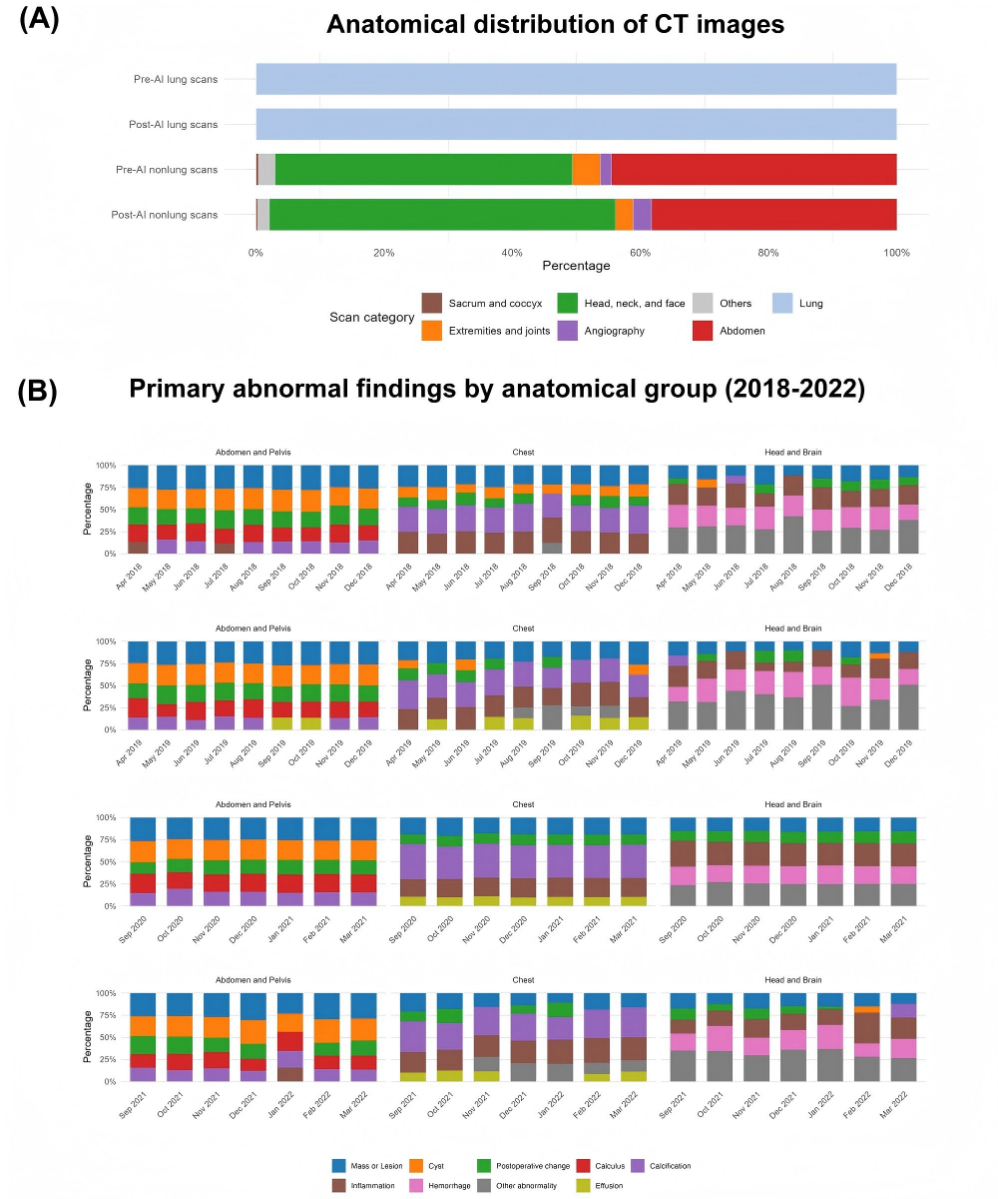
Anatomical distribution of scans and prevalence of key abnormal findings. (A) Bar chart showing the anatomical distribution of computed tomography (CT) images for the chest (treatment) and nonchest (control) groups before and after the implementation of artificial intelligence (AI). (B) Stacked area charts depicting the monthly prevalence of primary abnormal findings from 2018 to 2021 for the 3 highest-volume anatomical groups: abdomen and pelvis (left), chest and lung (middle), and head and brain (right).

**Figure 3:**
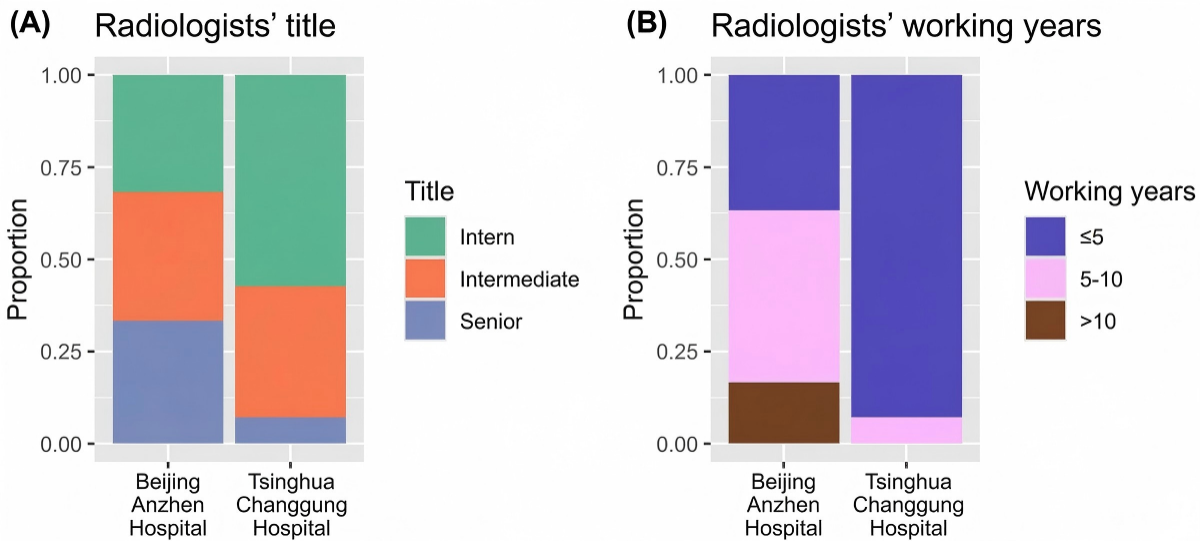
Radiologists’ job titles and working years by hospital.

We assessed the characteristics of the CT images across 2 key dimensions to ensure their comparability over time: case mix and case complexity. First, the anatomical distribution of CT images remained largely consistent, dominated by head and abdominal studies (Figure 2A). Second, the prevalence of primary abnormal findings (eg, “liver cyst” and “cerebral atrophy”), a proxy for interpretation complexity, was also longitudinally stable in high-volume domains such as abdomen and head CTs (Figure 2B). Given that the same 74 radiologists interpreted both intervention and control images across time, the overall case mix, case complexity, and workload remained stable. However, the job titles and working years varied for the radiologists (Figure 3). Those working at Beijing Anzhen Hospital had a higher job rank and longer working experience. Therefore, these variables were further adjusted in the main analyses. Multimedia Appendix 5 presents the outcomes of the parallel trend assessment, illustrating that the report-drafting times of both experimental and comparison groups displayed a consistent trend before the introduction of the AI system.

The adjusted DID model revealed a pooled effect size of 0.86 minutes (95% CI 0.14 to 1.57) after AI implementation. To assess the potential influence of anatomical site, we conducted sensitivity analyses by sequentially excluding each major category of nonchest CT images from the control group. As shown in Multimedia Appendix 6, the direction and magnitude of the AI effect remained generally consistent across all exclusion scenarios. However, this aggregated result should be interpreted with caution, as it is averaged over 2 hospitals with different implementation timelines. When breaking down to the hospital level, Tsinghua Changgung Hospital had a nonsignificant 0.90-minute increase (*P*=.12), while Beijing Anzhen Hospital saw a statistically significant 1.37-minute time saving (*P*<.001; Table 2). No statistically significant interaction was observed between AI exposure and radiologist seniority (all *P*>.09). In sensitivity analyses controlling for patient source, the estimated AI-associated effects remained robust (the overall DID estimate remained 0.86 minutes; *P*=.02).

**Table 2:** Adjusted report-drafting time differences for chest computed tomography (CT) vs others before and after artificial intelligence (AI) implementation, overall and by hospital.

			Intervention and group		Patients, n		Report-drafting time^a^ (minutes; 95% CI)		*P* value
**Overall (2 hospitals)**					185,044				
	**Control group^b^**								
		Pre-AI implementation		55,592		7.58 (6.32 to 8.85)		—^c^	
		Post-AI implementation		50,751		7.51 (6.10 to 8.93)		—	
		Pre-post difference		—		–0.07 (–0.62 to 0.48)		—	
	**Experimental group^d^**								
		Pre-AI implementation		38,278		6.79 (5.40 to 8.19)		—	
		Post-AI implementation		40,423		7.58 (6.17 to 8.99)		—	
		Pre-post difference		—		0.78 (0.26 to 1.30)		—	
DID^e^					—		0.86 (0.14 to 1.57)		.02
**Tsinghua Changgung**									
	DID			87,678		0.90 (–0.28 to 2.08)		.12	
**Beijing Anzhen**									
	DID			97,366		–1.37 (–2.04 to –0.70)		<.001	

^a^Report-drafting times are averaged over radiologists’ sex, working years, and job title.

^b^Control group refers to CT image reports of nonlung sites that were not subject to the launch of the AI system.

^c^Not applicable.

^d^Experimental group refers to lung CT image reporting, which could be affected by AI assistance.

^e^DID: difference-in-differences (comparing changes in report-drafting time for lung vs nonlung CT images before and after AI implementation).

**Figure 4:**
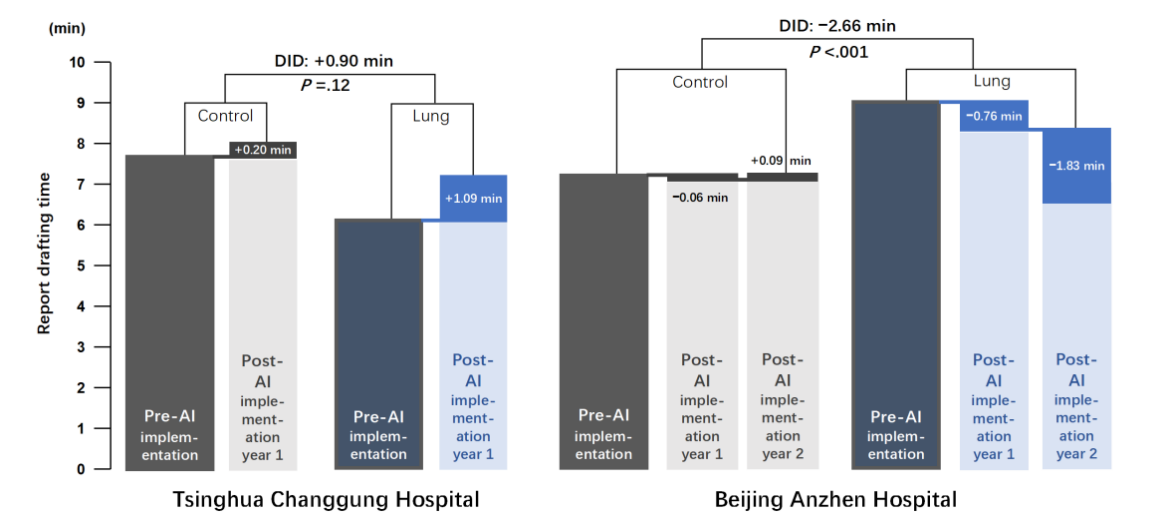
Adjusted changes in report-drafting time before and after artificial intelligence (AI) implementation. Gray indicates phases without AI assistance, and blue indicates phases with AI assistance. Tsinghua Changgung Hospital does not have second-year post-AI implementation data because the system was adopted later at that site. Report-drafting times are averaged over radiologist sex, years in practice, and job title. Difference-in-differences estimates compare changes in report drafting time for lung vs nonlung computed tomography reports before and after AI implementation. DID: difference-in-differences.

Subgroup analyses were conducted to account for the distinct implementation timelines of the AI systems at the 2 hospitals, as summarized in Figure 4 and Table 3. At Tsinghua Changgung Hospital, both the control group and the experimental group experienced extended report-drafting time, with a 0.90 minute (95% CI –0.28 to 2.08) nonsignificant increase attributable to AI assistance. However, Beijing Anzhen Hospital demonstrated an absolute 0.76-minute reduction in the AI-assisted group by the first year and a further 1.83-minute reduction by the second year. Using a DID framework, this corresponded to a 2.66-minute relative improvement vs the counterfactual trend (*P*<.001) 2 years into the AI implementation.

**Table 3:** Differences in adjusted report-drafting time comparing chest computed tomography (CT) vs others by post–artificial intelligence (AI) adoption duration.

			Adjusted report-drafting time^a^ before AI implementation (min; 95% CI)		Adjusted report-drafting time^a^ in the first year after AI implementation (min; 95% CI)		Adjusted report-drafting time^a^ in the second year after AI implementation (min; 95 % CI)
**Tsinghua Changgung**							
	Control group^b^	7.70 (6.61 to 8.79)		7.90 (6.26 to 9.53)		—^c^	
	Experimental group^d^	6.09 (4.69 to 7.59)		7.18 (5.38 to 8.99)		—	
	DID^e^	Reference		0.90 (–0.28 to 2.08)		—	
**Beijing Anzhen**							
	Control group^b^	7.36 (5.91 to 8.81)		7.30 (5.78 to 8.82)		7.39 (5.22 to 9.56)	
	Experimental group	9.22 (7.82 to 10.63)		8.46 (6.97 to 9.95)		6.63 (4.75 to 8.51)	
	DID	Reference		–0.70 (–1.35 to –0.05)		−2.66 (−3.35 to −1.96)	

^a^Report-drafting times are averaged over radiologists’ sex, working years, and job title.

^b^Control group refers to CT image reports of nonlung sites that were not subject to the launch of the AI system.

^c^Not available.

^d^Experimental group refers to lung CT image reporting, which could be affected by AI assistance.

^d^DID: difference-in-differences (comparing changes in report-drafting time for lung vs nonlung CT images before and after AI implementation).

## Discussion

### Adaptation of Diagnostic AI Systems in the Early Stages

Our findings indicate that the implementation of the AI system for lung nodule diagnostics was initially associated with an increase in report-drafting time, particularly in the first year of use. This transitional phase likely reflects a necessary adaptation period, as radiologists familiarized themselves with the system and learned to integrate it into existing workflows.

Technical barriers, such as outdated hospital IT infrastructure and limited interoperability with electronic medical record systems, may also impede seamless AI integration [23]. A potentially more important factor influencing the short-term impact of the AI system on lung nodule diagnosis may be physicians’ self-efficacy. In the initial stages of the AI system’s implementation, radiologists, especially junior staff, often lacked previous experience working with the AI system and were cautious in interpreting or relying on AI-generated results. This could partially explain the initial reporting delays observed at Tsinghua Changgung Hospital compared with the more experienced radiology team at Beijing Anzhen Hospital. Previous studies have shown that when radiologists lack confidence in the AI system’s capabilities or have not yet fully acclimated to its operation, they might spend additional time validating AI outputs, rechecking discordant findings, or temporarily reverting to traditional workflows. This transitional phase can offset the efficiency gains intended by the AI system [24,25]. Limited formal training, reliance on trial-and-error learning, and incomplete understanding of deep learning mechanisms can further undermine early trust and efficiency [26-28].

However, over time, these early barriers diminished with routine use. A previous survey conducted at the same 2 hospitals showed strong user acceptance: radiologists rated the AI system highly in usability (System Usability Scale score: mean 74.3, SD 11.9) and efficiency improvement (mean 4.6, SD 0.6 out of 5) [29]. This growing user confidence likely reduced the need for repeated result validation and enabled more streamlined integration of the system, ultimately contributing to the substantial efficiency gains observed in later stages, such as the significant reduction in report-drafting time during the second year at Beijing Anzhen Hospital.

A related observation at Tsinghua Changgung Hospital further supports the interpretation of early-stage friction. During the first year, the control group did not exhibit a statistically significant change in report-drafting time, whereas the AI group experienced a meaningful increase. We confirmed with the hospital that no workflow reorganization, software upgrades, or staffing changes occurred during the study period. Therefore, the relative inefficiency observed in the AI arm at Tsinghua Changgung Hospital is unlikely to reflect systemic improvements in the control arm; instead, it aligns with our proposed mechanism that AI systems initially introduce cognitive load and workflow disruption. In contrast, Beijing Anzhen Hospital did not demonstrate a comparable degree of early inefficiency among its AI users. We hypothesize that this might be due to the higher baseline experience and seniority of its radiologists (Figure 3), which may facilitate faster adaptation to AI-assisted workflows.

These findings underscore the importance of considering the user characteristics and testing for potential learning curve when evaluating the impact of AI systems. Early performance metrics may underestimate their long-term value if insufficient time is allowed for user adaptation. If the initial learning curve does exist, structured training and supportive onboarding environments can help accelerate this process and maximize clinical benefits [30].

### Long-Term Benefits of Diagnostic AI Systems

The long-term benefits of AI for diagnostic efficiency are complex and may vary significantly across institutions. While the pooled analysis suggests an overall increase in report-drafting time, this averaged effect masks the true clinical picture, where one hospital showed no immediate efficiency gains, while the other demonstrated a significant reduction in report-drafting time.

In high-volume radiology departments where radiologists face overwhelmingly heavy workloads over prolonged periods [31], information overload can lead to diagnostic errors [32,33]. Under such circumstances, even modest efficiency gains are valuable. In our study, extended use of the AI system for lung nodule analysis was associated with a reduction in average report-drafting time of up to 28% at one site. The AI system’s inherent capacity to autonomously identify and measure lesions markedly reduces the burden of manual labor while also expediting the generation of standardized text reports. These collective features of the AI system, in turn, reduce the necessity of repetitive writing tasks [18]. In settings where radiologists typically read more than 50 scans per day, such an improvement amounts to 2 hours of saved time per radiologist daily. This could allow for higher throughput, shorter patient waiting times, and more time allocated to complex cases.

Notably, these efficiency gains emerged alongside previously established diagnostic benefits. Our previous work evaluating the same AI system demonstrated improved lung nodule detection with a modest early rise in false positives, underscoring the need for training and adaptation [34]. That analysis further showed stable diagnostic accuracy after senior review, suggesting no substantial downstream burden attributable to AI-assisted drafting. These findings provide important clinical context for this study, which shifts the focus from diagnostic performance to operational efficiency. While this analysis focuses on first-round report drafting by junior radiologists, the combined evidence suggests that AI adoption does not merely shift workload upstream but could integrate efficiently into routine clinical workflows.

### Future Directions

While our study demonstrates the potential of AI to enhance efficiency within high-volume tertiary hospitals, its findings represent a necessary first step rather than a final answer. The successful deployment in these settings provides a proof of concept, but the ultimate goal is to improve health care resource allocation across the entire system. In terms of application stages, future mixed methods investigations are necessary to identify institutional and user-level factors that may shape AI adoption trajectories. With regard to clinical settings, future research must directly examine AI’s applicability in resource-constrained environments, which are often the hardest hit by medical shortages; first, partnering with primary and county-level hospitals as they begin to adopt AI; second, investigating the distinct barriers to adoption and effectiveness in low-resource settings (eg, outdated IT infrastructure, insufficient training, and different disease mixes); and third, examining how AI might be repurposed for tasks such as fast triage or ruling out common conditions in primary care, rather than for handling complex cases. Furthermore, the evaluation and validation of AI systems represent an iterative and dynamic process, necessitating a life cycle–based regulatory framework [35,36].

### Strengths and Limitations

Our study has several strengths. First, we conducted a longitudinal analysis of data from 2 tertiary hospitals in China, covering both pre- and post-AI implementation periods in real-world settings. The inclusion of a larger sample size enhances the reliability of our conclusions. Second, we applied an adjusted DID design, a quasi-experimental approach increasingly used in health services research, which strengthened the rigor of examining the causal effect of AI’s clinical impact. Finally, we analyzed report-drafting times by the same radiologists who interpreted both chest and nonchest CTs across the entire study period. This ensured that seasonal workload shocks, if any, would be shared across modalities within individuals, thus not differentially impacting the experimental and the control arm.

Nevertheless, our study had several limitations. First, both hospitals were large tertiary urban hospitals with high workloads and complex cases. As a result, our findings are considered only proof of concept, suggesting AI’s potential to alleviate efficiency bottlenecks in saturated diagnostic workflows. Our conclusions may not generalize to primary or rural hospitals with different infrastructural and caseload challenges. Second, although we conducted extensive sensitivity analyses to confirm longitudinal stability in case mix, case complexity, and preintervention trends, the use of nonchest CT examinations as controls during a pandemic-affected period may only serve as imperfect controls. While we adjusted for key physician-level characteristics, the DID model may have omitted unmeasured confounders. However, the exclusion of unstable periods, inclusion of hospital and seasonal fixed effects, and the staggered rollout design lend support to the internal validity of our results. Third, while the different AI vendors deployed at the 2 hospitals had comparable diagnostic functions, variation in their workflows and interfaces may have affected the drafting time. Finally, implementing a triple-difference design was not feasible in this study. We concur that future research incorporating data from nonadopting or primary care sites is a critical next step.

### Conclusions

Our findings highlight the heterogeneous impact of diagnostic AI on radiologists’ efficiency. While short-term implementation was associated with an increase in report-drafting time in one hospital with a shorter follow-up period, sustained use was associated with significant efficiency gains in another hospital after more than 2 years of adoption. These divergent outcomes underscore that the realization of AI-driven efficiency is not guaranteed but is critically contingent on site-specific implementation dynamics and local workflow integration. Therefore, adequate adaptation and education are necessary for AI systems to meaningfully alleviate radiologists’ workloads in the long run.

## Appendix

Multimedia Appendix 1 User interface of the artificial intelligence–assisted system under study.

Multimedia Appendix 2 Representative photographs of the artificial intelligence–assisted computed tomography reporting workflow.

Multimedia Appendix 3 Comparison of artificial intelligence system use workflows between the 2 hospitals.

Multimedia Appendix 4 Sample dataset.

Multimedia Appendix 5 Parallel trend assessment.

Multimedia Appendix 6 Sensitivity analysis using the leave-one-category-out approach.

## Abbreviations

AI: artificial intelligence
CT: computed tomography
DID: difference-in-differences
IRB: institutional review board

## Data Availability

The datasets generated or analyzed during this study are available from the lead and the corresponding author on reasonable request. A sample dataset with the same structure as the original data, but with anonymized, masked, and sequence-perturbed entries for privacy protection, is provided in Multimedia Appendix 4.
