# Supplementary figures and images for: Measuring the Impact of AI on Report-Drafting Efficiency in Chest Computed Tomography Interpretation: Retrospective Analysis
Source: J Med Internet Res. 2026 Mar 27;28:e77967. doi: 10.2196/77967 (PMC13069373; doi:10.2196/77967)

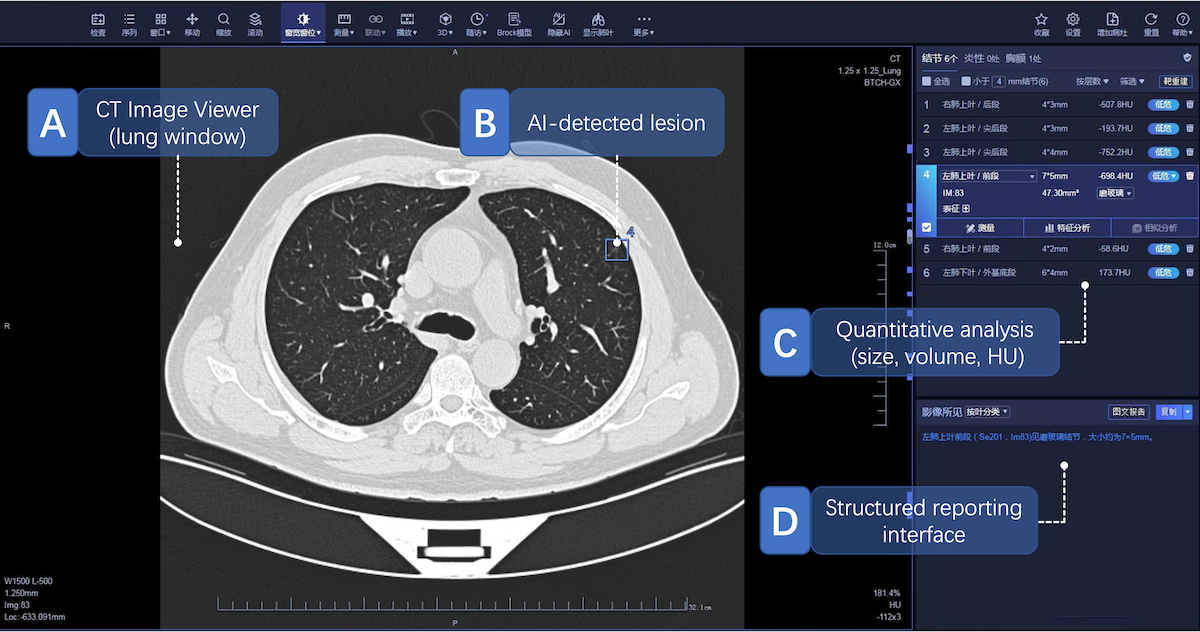

Supplement: Multimedia Appendix 1 [file jmir_v28i1e77967_app1.png]

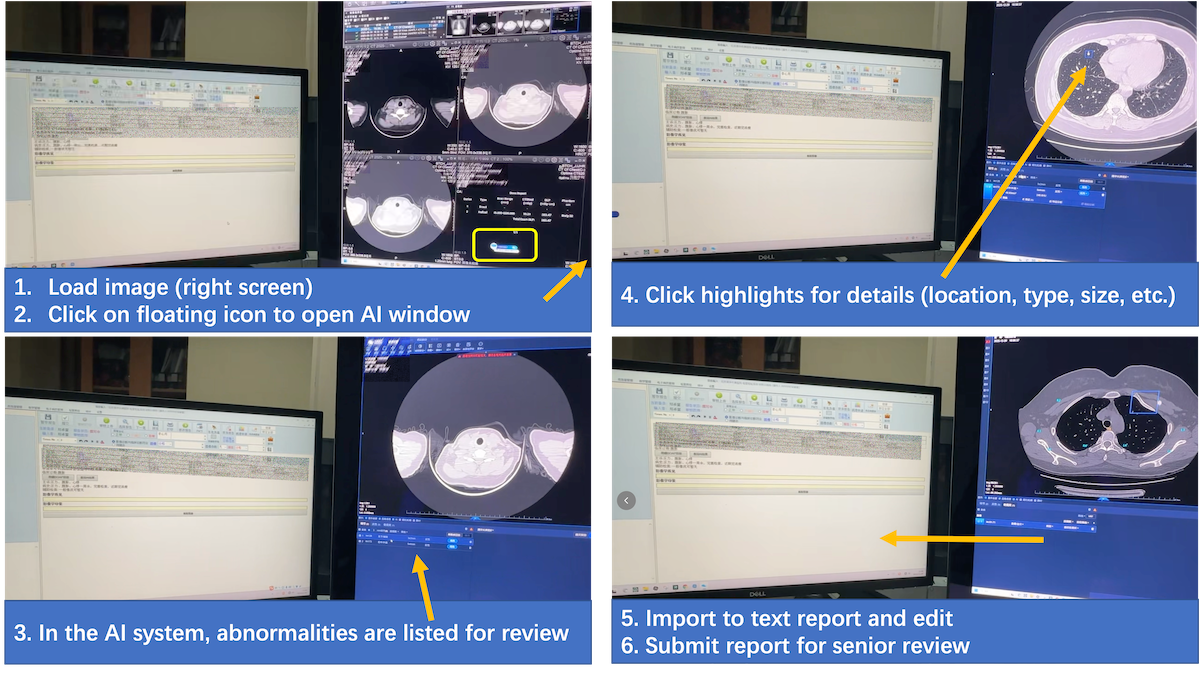

Supplement: Multimedia Appendix 2 [file jmir_v28i1e77967_app2.png]

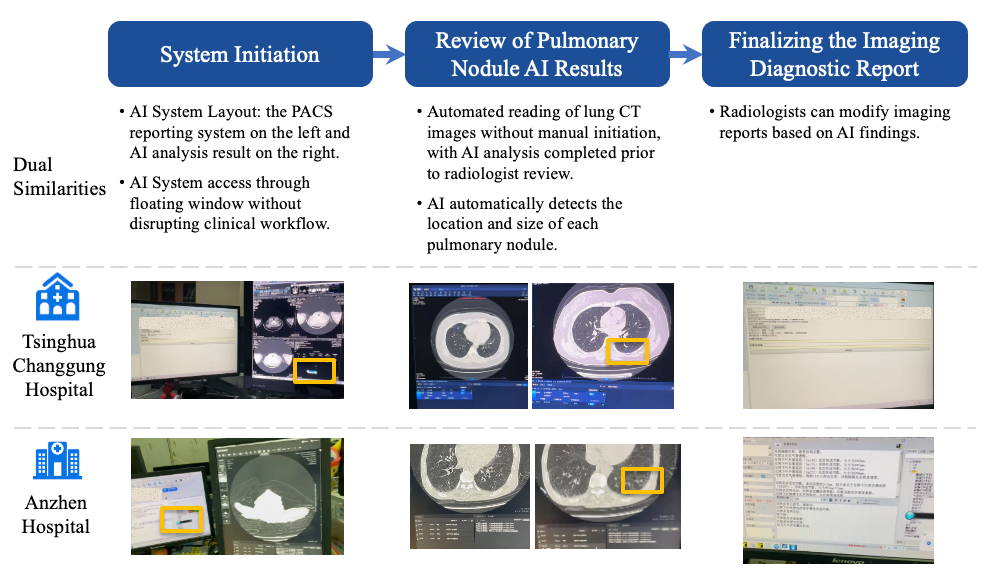

Supplement: Multimedia Appendix 3 [file jmir_v28i1e77967_app3.png]

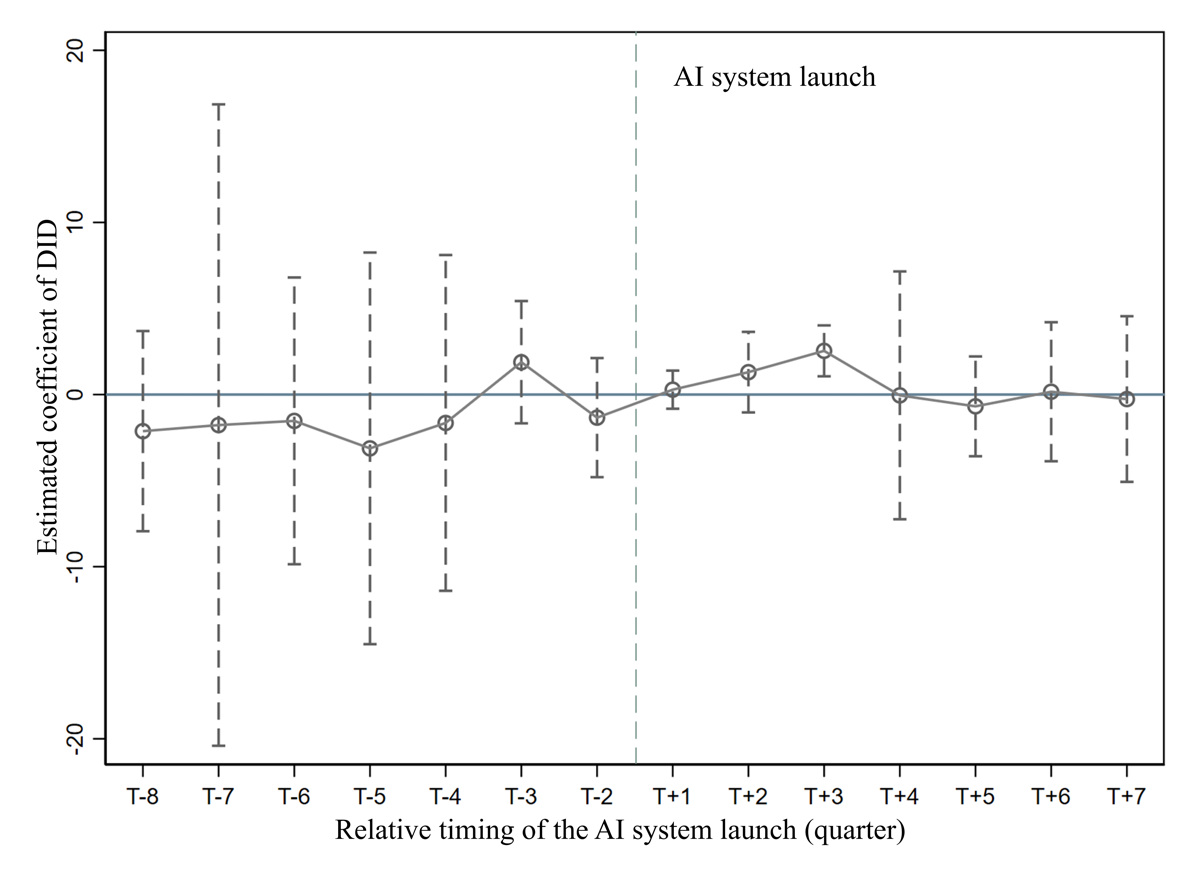

Supplement: Multimedia Appendix 5 [file jmir_v28i1e77967_app5.png]

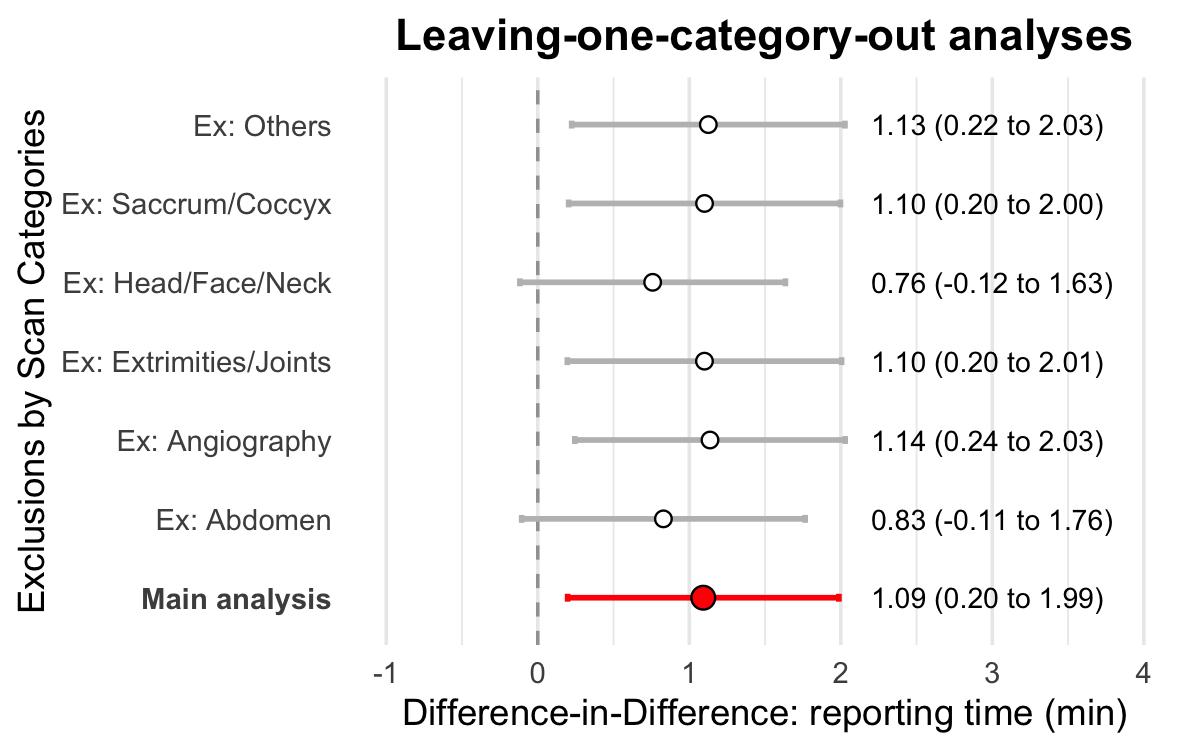

Supplement: Multimedia Appendix 6 [file jmir_v28i1e77967_app6.png]
